# Renal oncocytoma: a comparative clinicopathologic study and fluorescent in-situ hybridization analysis of 73 cases with long-term follow-up

**DOI:** 10.1186/1746-1596-5-32

**Published:** 2010-05-24

**Authors:** Marie Dvorakova, Rajiv Dhir, Sheldon I Bastacky, Kathleen M Cieply, Marie B Acquafondata, Carol R Sherer, Tracy L Mercuri, Anil V Parwani

**Affiliations:** 1 Department of Pathology, UPMC Presbyterian, 3550 Terrace Street, Pittsburgh, PA 15261, USA; 2 Department of Pathology, UPMC Shadyside, 5230 Centre Avenue, Pittsburgh, PA 15232, USA; 3 Insitu Laboratory, UPMC Montefiore, 3459 Fifth Avenue, Pittsburgh, PA 15213, USA

## Abstract

Clinical studies have confirmed that renal oncocytoma (RO) is a benign neoplasm with excellent prognosis. In diagnostically challenging cases of renal oncocytic epithelial neoplasms, fluorescent in-situ hybridization (FISH) is increasingly being used and its ability to distinguish RO from chromophobe renal cell carcinoma (ChRCC) has been documented. In this study, we evaluated the differential diagnostic contribution of FISH in cases of RO.

Clinicopathologic data and glass slides from 73 patients with RO were reviewed; 20 cases of ChRCC were included for comparison. FISH analysis of formalin-fixed, paraffin-embedded sections was performed using centromeric probes for chromosomes 1, 2, 7 and 17. FISH analysis revealed ROs had frequent loss of signal for chromosome 1 (56%) and 17 (44%). Tumors with more than one loss were common (41%) and 10% cases showed loss of all chromosomes examined. A total of 18% cases did not show any abnormality.

Our study shows that chromosomal abnormalities in both ROs and ChRCCs are common with frequent loss of chromosomes 1 and 17. No association was found between overall patient survival and the extent of chromosomal abnormalities. FISH results, even those showing significant chromosomal abnormalities, should not alter the primarily morphology-based diagnosis of RO.

## Introduction

Renal oncocytomas (ROs) comprise approximately 3-7% of renal epithelial neoplasms [1-3]. Common origin of RO and chromophobe renal cell carcinoma (ChRCC) from the intercalated cells of collecting ducts explains the histomorphologic, immunophenotypical, ultrastructural and molecular similarities of both neoplasms [2-7]. The benign nature of ROs, compared with the potential of ChRCCs for metastases and sarcomatoid transformation [8,9] has led to search for a confirmatory diagnostic modality; a broad array of ancillary studies have been employed in the differential diagnosis of the two neoplasms, including electron microscopy, flow cytometry, immunohistochemistry, cytogenetics and fluorescent in situ hybridization (FISH).

Cytogenetic studies of RO revealed genetic heterogeneity of these neoplasms; three distinct subsets were identified: 1) translocation between 11q13 and other chromosomes, 2) loss of 1/1p followed by loss of chromosome Y or 14 and 3) non-recurrent or no detectable aberrations [10]. Molecular and fluorescent in-situ hybridization (FISH) studies of ROs described partial or total loss of chromosome 1 to be the most consistently reported clonal chromosomal abnormality in RO [11-13] with a possible tumor suppressor gene expected on chromosome 1p [14]. In contrast, ChRCC was consistently found to display multiple losses of chromosomes 1, 2, 6, 10, 17 and 21 [12,15-17]. FISH was considered to be a potentially useful tool for distinguishing RO from ChRCC, with the former showing no abnormality or loss of chromosome 1 and the latter usually multiple additional chromosomal aberrations [12,18].

Based on the current data on genetic abnormalities occurring in ROs, we have employed an abbreviated FISH panel for chromosomes 1, 2, 7 and 17 in our institution that would aid in their differential diagnosis. In this study, we present our experience with this FISH panel and its contribution in the differential diagnosis along with survival data of 73 patients with RO.

## Materials and methods

### Case selection

The pathology records of the University of Pittsburgh were reviewed to find all patients who underwent primary nephrectomy for renal oncocytoma in the period of 25 years (1981 - 2006). Seventy-three cases of renal oncocytoma were selected from the UPMC archives using conventional histologic examination by two pathologists. For each patient, the data from the final surgical pathology report as well as from the clinical records were retrieved. In addition, 20 cases of ChRCC (11 cases of classic and 9 cases of eosinophilic variant) were included for comparison. Since the previously published FISH results did not show significant difference in observed abnormalities between the classic and eosinophilic variant of ChRCC [12], the two subtypes were not distinguished further in our study. Five (5) non-neoplastic kidney tissue sections were evaluated as a negative control.

### Fluorescent in-situ hybridization

Formalin-fixed paraffin-embedded sections were mounted and serially sectioned at 5-mm intervals. H&E section was used by the pathologist to determine the area of the tissue to be targeted for analysis. FISH slides were deparaffinized in xylene twice for 10 minutes, dehydrated twice with 100% ethanol and then pretreated using the Vysis Paraffin Pretreatment Kit. Slides were digested for 18 minutes in protease solution (0.5 mg/ml) at 37°C. FISH was performed using CEP1, CEP2, CEP7 and CEP17 centromere probes (Vysis, Inc., Downers Grove, IL). The target slide was denatured at 75°C for 5 minutes and dehydrated in 70%, 85%, 100% ethanol. Slides were incubated with probe overnight at 42°C in a humidified chamber. Post-hybridization washes were performed using 0.4× SSC/0.3% Igepal (Sigma) at 72°C for 2 minutes, followed by a room temperature 2 × SSC/0.1% Igepal wash for 30 seconds. Slides were air-dried in the dark and counterstained with DAPI (Vysis, Inc). Analysis was performed using a Nikon Optiphot-2 (Nikon, Inc) and Quips Genetic Workstation equipped with Chroma Technology 83000 filter set with single band excitors for Texas Red/Rhodamine, FITC, DAPI (UV 360 nm) (Vysis, Inc). Only individual and well delineated cells were scored, overlapping cells were excluded from the analysis. At least 60 cells were analyzed in the targeted region. Signal loss was considered significant if present in more than 30% of cells, gain if present in more than 20% of cells examined.

## Results

### Clinico-pathological characteristics of the study population with clinical follow-up

In the RO group, the study population included 51 males and 22 females (male to female ratio 2.3:1) with an average age of 67 (range 29 - 83) years. A total of 32 patients 44%) underwent partial nephrectomy (nephron sparing surgery), the remaining 41 patients (56%) radical nephrectomy with or without ipsilateral adrenalectomy. The mean tumor size was 4.0 cm (range from 1 - 21 cm). Small vessel invasion was present in 4 (5.5%) cases, and invasion into perinephric adipose tissue in 5 (6.8%) cases. None of the patients had evidence of metastatic disease at diagnosis. After a mean follow-up of 64 months (range from 1 to 293 months), a total of 46 (63%) of patients were alive with no evidence of disease; 14 (19%) had died of other causes and no follow up was available for 13 (18%) patients. None of the patients had disease recurrence nor developed metastases. The control ChRCC group consisted of 11 males and 9 females male to female ratio 1.2:1) with a mean age of 64 (range 35 - 88) years (Table 1). Average tumor size was 6.1 cm (range 1 - 14.5 cm). A total of 8 patients (40%) were treated with partial nephrectomy, 12 (60%) with radical nephrectomy. Small vessel invasion was present in 3 (15%) cases, and invasion into perinephric adipose tissue in 2 (10%) cases; one patient had tumor grossly extending into the renal vein. After a mean follow up of 60 months (range from 25 - 87 months), one patient with a tumor extending into the perinephric adipose tissue developed local recurrence 25 months after a radical nephrectomy. Similarly to the renal oncocytoma group, none of the patients with available follow-up died of disease or developed metastases.

**Figure 1:**
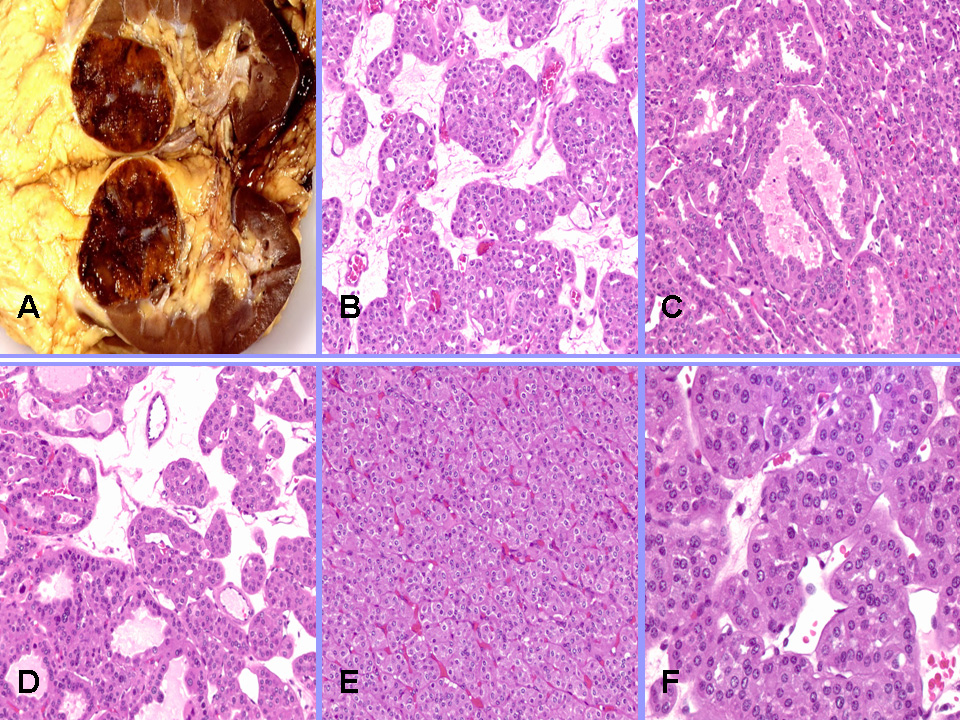
**A. Characteristic gross appearance of renal oncocytoma - well circumscribed, mahogany brown tumor with a central scar**. Histology: B. Typical oncocytoma composed of nests and acini of polygonal to round oncocytes with abundant densely granular eosinophilic cytoplasm and round nuclei embedded in loose myxoid or hyalinized stroma surrounded by a delicate reticulin framework (classic organoid pattern). C. Closely packed cystically dilated tubules seen in the tubulocystic pattern. D. Mixed pattern. E. Solid pattern with compactly arranged nests or sheets of oncocytes posed the biggest diagnostic challenge, especially in cases with its predominance. F. Typical oncocytes with abundant granular eosinophilic cytoplasm and round nuclei with smooth contours and small nucleoli.

**Figure 2:**
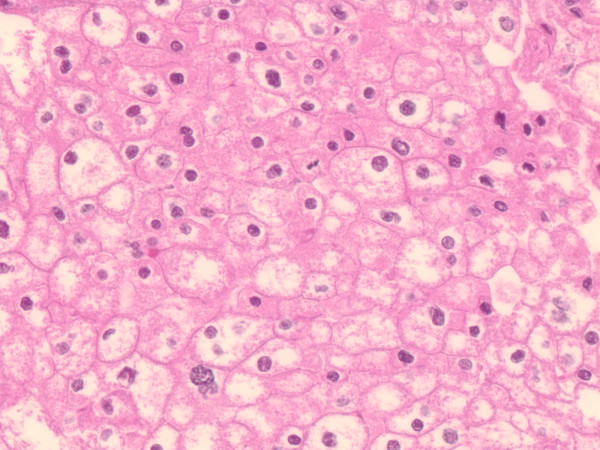
**Classic ChRCC composed of solid sheets, nests, acini or broad trabeculae of large polygonal cells with distinct cell borders and abundant, reticular, variably eosinophilic cytoplasm and irregular nuclei with wrinkled nuclear membrane and perinuclear halos**.

Characteristic histological features of both RO and ChRCC were observed (Figures 1 and 2). ROs exhibited a mix of architectural patterns; most commonly the "classic" organoid pattern with nests of polygonal to round oncocytes with abundant densely granular eosinophilic cytoplasm and round nuclei embedded in loose myxoid or hyalinized stroma. Other patterns were also seen (Figure 1). ChRCCs showed compact architecture of solid sheets, nests, acini or broad trabeculae composed of large polygonal cells with distinct cell borders, abundant reticular cytoplasm, irregular nuclei with wrinkled nuclear membrane and frequent binucleation (Figure 2).

In 32 cases, the initial diagnosis of renal oncocytoma was based on histology alone (44%); a total of 41 (56%) cases had some ancillary studies, such as electron microscopy (2 cases, 3%), DNA ploidy analysis (3 cases, 4%), FISH (24 cases, 33%) or histochemistry for Hale's colloidal iron (12 cases, 16%). A total of 68 cases (93%) were initially diagnosed as oncocytoma; however, 5 cases (7%) were diagnosed using descriptive terms such as "atypical oncocytic neoplasm" or "renal cell carcinoma of oncocytic type (oncocytoma)". In contrast, all of the chromophobe renal cell carcinomas were initially worked-up using ancillary studies such as Hale's colloidal iron histochemical stain (19/20 cases, 95%) or FISH (12/20 cases, 60%).

### FISH analysis

**Figure 3:**
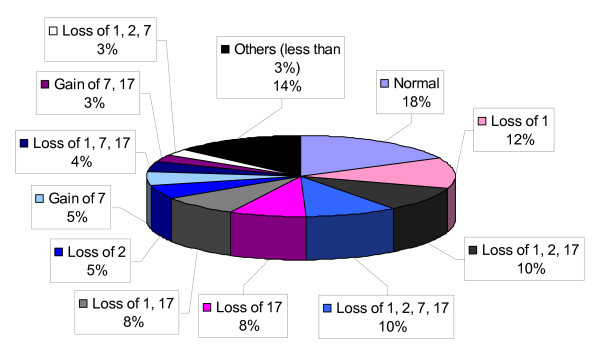
**Spectrum of chromosomal abnormalities in renal oncocytomas observed in our study using fluorescent in-situ hybridization panel**. In addition to isolated loss of chromosome 1, a wide variety of combined losses was detected. The previously described chromosomal profile (no abnormalities or isolated loss of chromosome 1) could possibly distinguish renal oncocytoma from ChRCC; however, these were seen in only 30% of renal oncocytomas in our study. In contrary, one of the ChRCCs with typical morphology and positivity for Hale's colloidal iron showed no chromosomal abnormalities.

**Figure 4:**
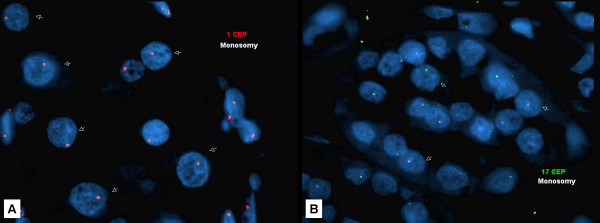
**Most common chromosomal abnormalities in renal oncocytoma observed in the current study using fluorescent in-situ hybridization analysis**: A - Loss of chromosome 1, B - Loss of chromosome 17.

The FISH results are summarized in Table 2 and Figure 3. Tumors with more than one loss were common (30/73, 41%) and 7/73 (10%) cases showed loss of one signal in all chromosomes examined. A total of 13 (18%) cases did not show significant losses or gains when compared to normal renal tissue. Loss of chromosome 1 (41/73, 56%) and 17 (32/73, 44%) were the most common abnormality (Figure 4); isolated loss of chromosome 1, however, was observed in only 8/73 (11%) of ROs.

ChRCCs consistently showed multiple chromosomal abnormalities, and 6/20 (30%) of cases were abnormal in all chromosomes examined (Table 2, Figure 3). The one case with a completely normal FISH analysis was a case of eosinophilic chromophobe renal cell carcinoma with a Fuhrman nuclear grade 2 and a strong cytoplasmic positivity for Hale's colloidal iron. None of our negative control sections from the adjacent non-neoplastic renal tissue showed any losses or gains exceeding the 30 and 20% threshold, respectively.

## Discussion

Renal oncocytomas are relatively uncommon neoplasms accounting for 3-7% of primary renal epithelial neoplasms [1-3]. The majority of RO has distinctive cytoarchitectural features that permit accurate diagnosis; however, the occasional differential diagnostic challenge between RO and ChRCC has been repeatedly acknowledged in the literature [2,3,18,19]. Both renal oncocytoma and chromophobe renal cell carcinoma originate form the collecting duct intercalated cells and can therefore share similar histomorphologic, immunophenotypical and ultrastructural characteristics [2-5], as well as gene expression profiles [6,7,20]. Renal oncocytomas can contain foci of atypical cells and show vascular space invasion or extracapsular extension not adversely affecting the overall excellent prognosis of this neoplasm [2,3]; the metastatic potential of RO remains controversial [21].

Although ChRCC was found to have low metastatic potential and its prognosis and long term survival is considered to be excellent [22], ChRCC was also found to have a potential to recur, metastasize and undergo sarcomatoid transformation [8,9]. The difficulty to distinguish the two neoplasms has been demonstrated by the broad array of ancillary studies employed so far routinely in the differential diagnosis such as electron microscopy, DNA flow cytometry, immunohistochemistry, cytogenetics, and FISH. A small proportion of renal epithelial neoplasms with eosinophilic cytoplasm cannot be definitively classified based on histology alone [19].

Based on published cytogenetic studies, ROs are genetically heterogeneous tumors. Fűzesi et.al proposed subclassification into 3 groups: the first one defined by translocation between 11q13 and other chromosomes, the second with a loss of 1/1p followed by loss of chromosome Y or 14, and the third with non-recurrent or no detectable aberrations [10]. Prior FISH studies have detected a varying incidence of partial or total loss of chromosome 1 in RO ranging from 10 - 59% [11-13], whereas loss of other autosomes was not detected. In contrast, ChRCCs were consistently found to have multiple recurrent chromosomal abnormalities, such as losses of chromosomes 1, 2, 6, 10, 13, 17 and 21 by FISH, conventional cytogenetics and comparative genomic hybridization [12,15,16,18,22]. No significant difference in chromosomal abnormalities was found between the classic and eosinophilic variant of ChRCC [12]. Since both RO and ChRCC can show loss of chromosomes 1 and Y, it was hypothesized that a subset of ROs may progress to ChRCC with subsequent loss of chromosomes 2, 6, 10, 13, 17 and 21 [23].

Based on the above data, we have employed an abbreviated FISH panel for chromosomes 1, 2, 7 and 17 to guide us in the differential diagnosis of renal epithelial neoplasms with eosinophilic cytoplasm. Whereas an isolated loss of chromosome 1 would favor the diagnosis of RO, multiple losses of chromosomes 1, 2 and 17 were expected in ChRCC cases. Although abnormalities involving chromosome 7 are not specific and have been reported in other types of malignancies, we included this chromosome since its trisomy (together with trisomy 17) was observed in papillary renal cell carcinoma [24,25] that can rarely contribute to the pool of unclassifiable cases of renal epithelial neoplasms with eosinophilic cytoplasm.

In our current study, the incidence of chromosomal abnormalities in ROs (60/73 cases, 82%) was significantly higher than in the previous reports [10,12,18]. Brunelli et al. [12] analyzed 10 RO by FISH using centromeric probes for chromosomes 1, 2, 6, 10 and 17; only one case showed loss of signal for chromosome 1, and no other aberrations were detected. The criteria for chromosomal losses were similar (22 - 30% of cells with one signal vs. 30% in the current study); however, the number of nuclei examined in our study was lower (60 vs. 100 - 200), but sufficient for overall assessment. Only one similar case of an incidentally discovered RO with multiple chromosomal losses involving chromosomes 1, 2, 3, 6, 8, 9, 15, 17, 21 and 22 in a 55-year-old male was identified in the literature [26]. The second most common genetic alteration in our subset of RO was the loss of signal for chromosome 17 (32/73, 44%); a common finding reported in ChRCCs and also observed in the control ChRCC subset in our study. Recently, a tumor suppressor gene associated with Birt-Hogg-Dubé syndrome was mapped to the pericentromeric region of 17p chromosome [27-30]. Birt-Hogg-Dubé syndrome is a rare genodermatosis characterized by the development of dermatologic lesions (fibrofolliculomas, trichodiscomas, acrochordons), lung cysts with spontaneous pneumothorax, colonic polyps and also renal tumors, mainly hybrid oncocytic neoplasms with both ChRCC and RO morphology, ChRCCs, clear cell carcinomas, oncocytomas and papillary renal cell carcinomas [31].

A total of 3/73 RO cases showed loss of chromosome 1 and gain of chromosome 7 and/or 17; morphologically, 2 cases displayed characteristic alternating nested and organoid growth patterns; the third case had predominantly tubulocystic arrangement of typical oncocytes. All three were initially diagnosed as RO without any ancillary studies. Gains of chromosome 7 and/or 17 without any accompanying chromosomal loss was observed in 4/73 cases; histologic review confirmed the initial diagnosis of RO in all four cases; however, only some of the original histologic sections were available for review in 3 cases precluding a definitive diagnosis. RO cases with extracapsular or vascular space invasion usually showed multiple chromosomal losses or gains; however, one of the cases with perirenal soft tissue extension was normal by FISH. Long term follow-up of patients included in our study confirmed the excellent prognosis of RO as none of the patients died of disease, developed metastases or local recurrence. No association was found between overall patient survival and the extent of chromosomal abnormalities detected by FISH.

The incidence of chromosomal abnormalities in ChRCC was in agreement with previous studies [12,15,16]; 19/20 (95%) cases showed multiple losses involving chromosomes 1, 2 and 17 in different combinations; in addition, loss of chromosome 7 was observed in 6/20(30%) cases and 6/20 (30%) cases showed loss of signals for all chromosomes examined (Figure 2). A total of 3/20 (all classic ChRCCs positive for colloidal iron) had chromosomal losses associated with gains of chromosome 7 and/or 17. Only one case did not show any gains or losses; review of the slides revealed an eosinophilic variant of ChRCC with a strong cytoplasmic positivity for colloidal iron (Figure 3). Rare cases of ChRCCs with no chromosomal abnormality have been reported [12,16]. One patient in the control ChRCC group developed local recurrence 25 months after partial nephrectomy; the reminder of the patients was disease free or died of other causes.

Our FISH study of ROs, which is largest to date, was based on a series of well-defined oncocytomas diagnosed according to the current morphologic WHO classification criteria [1]. Our results show that chromosomal abnormalities in ROs are common and include frequent losses of chromosomes 1 and 17, yet another feature that overlaps with ChRCCs. FISH was not found to be a useful diagnostic modality in the differential diagnosis of RO and ChRCC and did not alter the initial primarily morphology-based diagnosis of renal oncocytoma in any of the 73 cases. Our results suggest that RO and ChRCC are genetically related tumors and support the hypothesis that they might rather represent a spectrum of neoplasia than two distinct neoplasms. This finding is further supported by the concurrent RO and ChRCC as well as their hybrid form in patients with Birt-Hogg-Dubé syndrome. The significance of specific combinations of chromosomal abnormalities as well as the impact on prognosis, propensity to metastasize, recur and undergo sarcomatoid transformation remains to be further investigated.

## Competing interests

The authors declare that they have no competing interests.

## Authors' contributions

MD carried out the research studies, participated in the drafting of the manuscript. KMC, CRS and TLM carried out the FISH studies. MBA carried out the immunohistochemical studies. RD and SIB participated in the design of the study. AVP conceived of the study, helped draft the manuscript and participated in its design and coordination. All authors read and approved the final manuscript.
